# Oral Aspects Identified in Atopic Dermatitis Patients: A Literature Review

**DOI:** 10.2174/1874210601812010424

**Published:** 2018-05-31

**Authors:** Aline Domingues Tavares Oliveira, Camila Stofella Sodré, Dennis de Carvalho Ferreira, Eliane de Dios Abad, Simone Saintive, Márcia Ribeiro, Fernanda Sampaio Cavalcante, Bruna Piciani, Lucio Souza Gonçalves

**Affiliations:** 1 Department of Microbiology, Federal University of Rio de Janeiro, Rio de Janeiro, Brazil; 2 Department of Pathology, Fluminense Federal University, Rio de Janeiro, Brazil; 3 Faculty of Dentistry, Estácio de Sá University, Rio de Janeiro, Brazil; 4 Department of Clinical Medicine, Federal University of Rio de Janeiro, Rio de Janeiro, Brazil; 5 Faculty of Dentistry, Veiga de Almeida University, Rio de Janeiro, Brazil; 6 Pediatric Dermatology Service, Federal University of Rio de Janeiro, Rio de Janeiro, Brazil; 7 Medical Genetics Service, Federal University of Rio de Janeiro, Rio de Janeiro, Brazil

**Keywords:** Atopic dermatitis, Oral manifestations, Dental caries, Xeroderma, Nasopharyngeal mucosa, Chronic inflammatory skin condition

## Abstract

**Introduction:**

Atopic dermatitis is a chronic inflammatory skin condition that is more prevalent in children (10-20% of the world's population) than in adults. As its etiology is multifactorial, it is important to know the most frequent oral manifestations in atopic dermatitis patients.

**Methodology:**

In the last decades, the correlation between atopic dermatitis and conditions and/or changes in the oral cavity has been demonstrated by several studies. The objective of this paper was to describe, through a review of the literature, the oral health conditions and/or oral aspects identified in patients with atopic dermatitis.

**Search Strategy:**

A descriptive literature review was carried out through a bibliographical survey based on the last 10 years, in order to answer the study questions.

**Results:**

As a result, we found six studies with different sample sizes, ranging from 43 to 468 patients, and the majority of them were of cross-sectional study design.

**Discussion:**

Two studies performed their analysis through dental exams and reported that patients with atopic dermatitis tend to have a greater frequency of carious lesions, and two studies correlated *Candida* with atopic dermatitis through mycological analyzes.

**Conclusion:**

There are a few studies in the literature that identify the oral aspects of atopic dermatitis. More investigations are needed in order to contribute to the knowledge of such oral aspects and the approach to treat these patients regarding oral health.

## 1 INTRODUCTION

Atopic Dermatitis (AD) is a chronic inflammatory skin condition that can be accompanied by asthma and other manifestations of atopy. Among the clinical features of AD are intense pruritus, xeroderma, and recurrent eczema, which may be acute, subacute or chronic [1-3]. There are periods of improvement and exacerbation, and such symptoms are often present in other family members [4-6].

AD diagnosis is clinical and is based on the criteria suggested by Hanifin and Hajka [7]**,** as there is no specific laboratory marker. Control of the disease and treatment of exacerbations is carried out by environmental control, avoiding triggering factors, such as dust and cooling air. Also, skin care and topical or systemic drug treatment depending on the severity of the condition can be used as preventing strategies. One of the main drugs used in dermatology are topical corticosteroids. However, due to the chronic and recurrent character of AD, immunosuppressive drugs, such as cyclosporine A, methotrexate, azathioprine, or mycophenolic acid are sometimes necessary [8].

Over the last few years, various studies have tried to identify factors that exacerbate AD. One example is the research related to AD and intestinal microbioma [9]. Another factor that has not been adequately addressed is the oral health of children with AD. This is still an issue, despite its importance.

Studies, since 1987, have demonstrated the correlation between atopic dermatitis and manifestations and/or changes in the oral cavity. For example, the study by Hannuksela and Väänänen [10] conducted a survey to determine the types and prevalence of malocclusions in a group of 217 7-year-old children, of which 82 children had symptoms of atopy. These authors observed that atopic dermatitis was correlated to posterior crossbite and atypical swallowing. Congruently, it is known that atopic diseases generally increase the frequency of class I occlusion and reduced overbite, and that the development of crossbite and other changes in the dentition may be associated with the fact that the nasopharyngeal mucosa is hyperreactive in children with AD [10].

In 2000, in a study with 24 patients with atopic dermatitis and 7 with other dermatological diseases, Yoshida and Amatsu investigated whether the dermatological manifestations in the face of patients with AD could reactivate the latent Herpes Simplex Virus Type 1 (HSV-1) in the trigeminal ganglion, causing an asymptomatic excretion of this virus in the oral cavity. Although the results were not conclusive, according to the authors, there is a possibility that HVS-1 in the oral cavity can infect the skin in other locations, since recurrent skin lesions may be a trigger for herpetic eczema in patients presenting recurrent episodes of eczema [11].

Some authors have reported that atopic patients with high serum IgE levels are more likely to present a Geographic Tongue (GT) [12-14]. One possible explanation for this is that psychosomatic factors and acute inflammatory conditions contribute to the emergence of both conditions. Although GT is not a specific feature of atopy, it is a condition that may be associated with atopic disorders and is often observed before the onset of atopy symptoms. The only study found in the literature that evaluated oral changes in patients with atopic dermatitis concluded that AD is not associated with GT [12-14]. The etiology of GT remains obscure, but it is known to have a chronic, inflammatory and immunomediated profile, with the involvement of cytokines. In view of these similarities with AD, further studies are needed to confirm whether there is a type of GT that is associated with AD [12-14].

Among some of the minor criteria for the diagnosis of atopic dermatitis established by Hanifin and Hajka [3] is atopic cheilitis, a condition that causes “redness” due to mechanical stress and environmental and physiological factors. Such manifestation creates difficulty to treat the site where there is a persistent defect of the epithelial barrier [15].

The prevalence of AD around the world has increased over the last 30 years and is now found in Africa, East Asia, Western Europe, parts of Northern Europe and has affected as much as 18% of the population in the United States [16, 17]. This increase may be due to changes in the pattern of environmental pollutants, a reduction in breastfeeding, an increase in sensitivity of the individuals involved, improved personal hygiene, a reduced risk of infections during childhood and consequently an increase in sensitivity to allergens, and in urbanization, with a higher prevalence of AD in urban settings [7]. The incidence of AD is higher in children (10-20%), than in adults where it is only 1-3% [8].

The diagnosis of AD is complex as there is still yet no effective specific laboratory marker, and only a pre-clinical diagnosis has been made, which is defined according to the primary and secondary criteria recommended by Hanifin and Hajka [3] and/or by Williams *et al.* [18]. The treatment is based on the exclusion of the environmental causes such as dust and weather conditions; and association with topical corticosteroids. However, due to factors such as frequent recurrence, rebound of the disease, due to the use of potent topical corticosteroids, the literature suggests new therapeutic alternatives for patients with AD [19]. Despite all the recent information found in the literature, the oral health of children with AD is still insufficiently addressed especially since certain important factors, concerning oral health, influence the daily life of patients with AD.

Chronic diseases such as atopic dermatitis usually have a multifactorial etiology, and therefore their diagnosis and clinical follow-up require a multidisciplinary professional approach in which dentistry plays a prominent role as the oral cavity frequently signals or evidences important manifestations of such diseases [20].

Since 1987, studies have demonstrated correlations between atopic dermatitis and possible manifestations/ alterations in the oral cavity such as an increased frequency of class I occlusion, reduced overbite, susceptibility to cariogenic activity, reactivation of the Herpes Simplex Virus type 1 (HSV-1), causing its release into the oral cavity, and the worsening of AD in patients with odontogenic focal infections. Moreover, the use of inhaled corticosteroids contributes to the development of candidiasis [10, 11,21-25].

Although there are few studies linking atopic dermatitis to oral aspects, this aspect of AD is clearly still a topic that should be investigated further. Therefore, the objective of this work was to describe, through a review of the literature, the oral health conditions and/or aspects identified in patients with atopic dermatitis using articles published over the last 10 years.

## 2 METHODOLOGY AND SEARCH STRATEGY

This research was carried out *via* a literature review [26]. This study came from a research project in atopic dermatitis that had an interdisciplinary microbiological and care practices approach that included oral health. The project aimed to promote care and quality of life for AD patients. The oral aspects were highlighted so that the (dental) professionals were more prepared to assist these patients and could formulate strategies of intervention. The following (guideline) questions were prepared to carry out the present review: What oral aspects were identified in the scientific literature as being related to patients with atopic dermatitis? Did these findings correlate with the severity of AD?

The bibliographical survey consisted primarily of five online databases: PubMed, the Scientific Electronic Library Online (SciELO), Scholar Google, Latin American and Caribbean Health Sciences (LilaCS), and the Brazilian Dentistry Library (BBO). As the search criteria, documents published and found in the “advanced search” mode were investigated, using cross-correlation with the following keywords: “atopic dermatitis”, “oral manifestations”, “mouth”, “oral pathology”, “dental caries” and “stomatognathic diseases” which were identified and selected according to the addressed subject. Also, the AND operator was used in these searches. These words were selected because they related to the topic of interest and were previously checked in the MeSH of Medline. Also, the terms “childhood” and “dental disorders” were included although they were not directly described in the MeSH.

The articles were selected from each database following the inclusion criteria: Title and Abstract of the article compatible and related to the topic of interest; published in full in the following languages: Portuguese and/or English; published and/or available online from January 2006 to January 2016; and based on original research and/or clinical case reports. Theses, books, dissertations, patents, literature reviews and articles whose theme was not related to the purpose of the study were excluded.

## 3 RESULTS

**Fig. (1):**
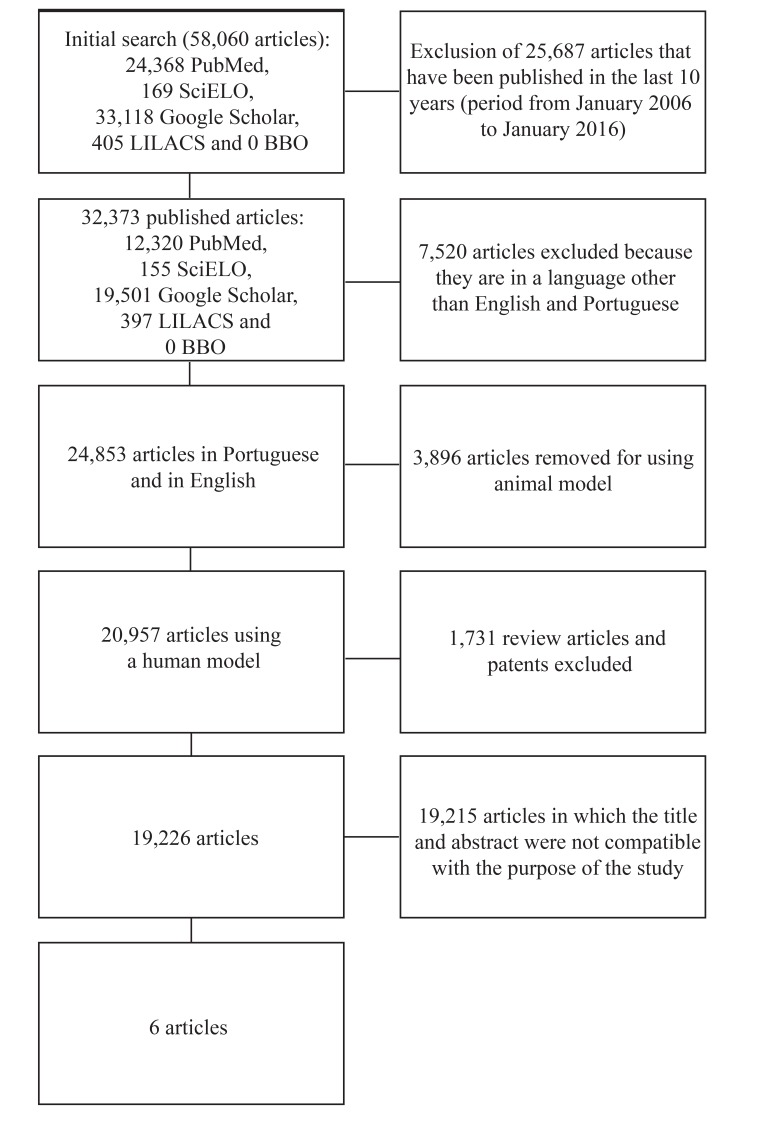
Article selection methodology.

The initial search for articles resulted in a total of 24,368 from PubMed articles, 169 articles from ScieLo, 33,188 from Google Academic, 405 from LilaCS and no articles from BBO (Fig. **1**). After applying the inclusion and exclusion criteria, 6 studies were selected for the review (Table **1**).

The sample size in the studies selected was very diverse, ranging from 43 to 468 patients. Most articles were observational, cross-sectional (5/6 studies) and all used the English language (6/6 articles). In relation to the severity criteria of AD, 2 studies [22, 27] used SCORAD as the criterion for severity of AD, while 2 other studies [21, 28] used EASI (Eczema Area and Severity Index) [3]. The other 2 studies [29, 30] did not assess the severity of AD. Yamaguchi *et al*. [29] used the mouth breathing criterion from a questionnaire carried out with pre-school children. In this case, atopic dermatitis was evaluated in conjunction with other conditions, such as asthma and allergic rhinitis, among others which were found using the questionnaire.

The microbiological analyzes of two studies [22, 27] detected the presence of *Candida albicans* with atopic dermatitis through mycological analyzes. The latter also used PCR for the D1/D2 domain of the ribosomal subunit gene (26S) and sequencing. Serum IgG, IgM and IgA antibodies were also detected by ELISA using *anti-C. albicans* antibodies.

The other studies followed different methodologies, such as evaluating the presence of root caries in 43 patients with chronic atopic dermatitis resistant to conventional therapy and who were reassessed 3 months after receiving dental treatment. In this study, the AD patients with an odontogenic focal infection showed a greater improvement in the EASI score after dental treatment in relation to the group that did not receive the dental treatment [21]. Another study evaluated salivary cortisol levels [28] demonstrating that the level of salivary cortisol in patients with AD was higher than in the control group.

Yamaguchi *et al.* [29] used a questionnaire to evaluate the relationship between mouth breathing and atopic dermatitis, in which the subjects were divided into three groups: patients with AD, asthma and allergic rhinitis. The results showed there were associations between AD and mouth breathing during the day and during sleep. Another study with 300 patients, 90 (30%) with AD, assessed the oral health conditions, through the presence of carious lesions and examination of the soft tissues. Here the results showed a high prevalence of medical history of spoil habit in 76.6% of the children and adolescents studied: thumb sucking (36 children), tongue protrusion (11 children), infantile swallowing (40 children), mouth breathing (17 children), onychophagia (15 children), alterations in the dental anatomy (13 children), agenesis (5 children), hypoplasias (7 children), dental caries (49 children) and dental malocclusions (58 children**)** [30].

## 4 DISCUSSION

Our group focuses on improving the quality of life for children with dermatological diseases, as well as scientific research in atopic dermatitis, in particular, which is one of the most frequent and disturbing diseases in childhood [20, 31]. The oral health of patients with AD is still an underexplored subject, although some studies have shown a correlation of AD with the presence of oral manifestations.

Igawa *et al.* [21] coined the term odontogenic focal infection to describe root caries in their study. These authors reported that this type of infection could contribute, based on previous studies that found a relationship between focal infections and psoriasis, to the worsening of atopic dermatitis. The authors evaluated 43 patients with chronic AD, who were resistant to conventional therapy. Although root caries (active/silent) were asymptomatic and were present in some patients, all the patients underwent radiographic examination and were treated with conventional therapy, and when necessary, dental treatment, including dental extractions, was carried out [20]. The results showed that the patients had a significant improvement in the severity of AD after 3 months of treatment. This improvement was especially noticeable in the group of patients who had root caries, and who obtained a significantly greater improvement than the control group. The total number of patients, in this study, who had carious lesions was much lower than the number of patients in the study by Perugia *et al.* [30] with 300 children and adolescents of both genders, of whom almost 90 had AD. The patients with AD had a high prevalence of carious lesions (54%) and dental malocclusion (64,4%), suggesting the role of an odontogenic focal infection or presence of carious lesions as possible AD aggravating factors [20, 30]. These same authors also described medical history of spoil habit (76.6%) such as thumb sucking, infantile swallowing and mouth breathing and dental alterations in 14.4% of the children, such as agenesis and hypoplasias.

Outpatients with moderate or severe AD are nearly always treated with corticosteroids, immunosuppressants/ immunomodulators, and antibiotics. However, these drugs, especially inhaled corticosteroids, favor the occurrence of Candidiasis, increasing the cariogenic activity due to the symbiosis relationship between *Streptococcus mutans* and *Candida albicans* [24].

Some liquid oral antibiotics also have cariogenic potential, as they contain components such as sugars and acids, which are added for the purpose of improving their taste, bioavailability and chemical stability [32]. The ability of an antibiotic to affect tooth enamel can be calculated mathematically by the Data Envelopment Analysis (DEA) methodology. This technique considers undesirable (high sugar concentration, low pH, high pH-titration and high viscosity) and desirable characteristics (absence of sugars, high pH, ​​low pH-titration and low viscosity) to evaluate the performance of the antibiotic. The performance can vary from 0 to 100% and the lower the value, the greater the probability of attack on dental enamel.

Currently, the most commonly prescribed antibiotic for pediatric patients with AD and with an infection is cephalexin [33]. First-generation cephalosporins, including cephalexin, are recommended in the main guidelines for the treatment of staphylococcal infections as first-line therapy. Moreover, this is because they are antimicrobials of good tolerability, low cost, are taken orally and they are active against both Gram-positive and Gram-negative bacteria [34]. The DEA methodology indicates that the performance of Cephalexin, in dosages of 500 Mg, is 62%. This value is associated with a greater possibility of dental enamel dissolution, allowing erosion and a risk of cariogenic activity. However, at a lower dosage of 250 mg, the antibiotic has a DEA percentage value of 100%, indicating a low probability of affecting tooth enamel [32].

In addition to the intrinsic characteristics of antibiotics, the relationship between xerostomia and salivary flow is another factor to be discussed in the cariogenicity of these drugs, due to the important function of the salivary flow in the prevention of caries. Drugs that cause a decrease in salivary flow include the antihistamines, which are often used by patients with atopic dermatitis. They have side effects on the Central Nervous System (CNS) and antimuscarinic effects including dry mouth. These effects occur due to the inhibition of the M3 receptors present in the salivary glands. Decreased salivary flow is conducive to cariogenic activity. Immunosuppressants, especially systemic and inhaled corticosteroids, are also commonly related to opportunistic oral infections. This is due to their suppressive action of inflammatory activity, which also prevents the action of T lymphocytes, macrophages and some enzymes, among others [35]. Thus, as already stated, *Candida albicans* is characterized as a commensal microorganism of the oral cavity of adults and children without presenting oral manifestations [36, 37]. However, in the presence of predisposing factors that may be present in AD, such as the use of broad-spectrum antibiotics, xerostomia and immunodeficiency; the pathogenic form of *Candida albicans* can lead to oral lesions [38-41].

Leibovici *et al.* [27] conducted a prospective study comparing the prevalence of *Candida* on the tongue of patients with psoriasis, AD patients and normal controls. In the study, the tests for different fungal species were performed, and the results showed that the prevalence of *Candida* on the tongues of patients with psoriasis was significantly greater (32%) than in AD patients (18%). However, Javad *et al.* [22] carried out a study with AD patients and healthy individuals to detect *Candida* colonization and to verify the *Candida*-specific humoral response in patients with AD. Oral cavity and skin samples were collected from the participants. *Candida albicans* was isolated from the skin and oral cavity of 23% of the AD patients, whereas only 6% of the control group presented *Candida albicans*. Unlike Leibovici *et al*. [24], Javad *et al.* [22] evaluated *Candida albicans*-specific IgG levels and observed significantly lower values ​​in AD patients compared to their controls.

Mouth breathing causes alterations or deficiencies in the saliva-mediated defense mechanisms of oral tissues, possibly resulting in a greater risk for the development of oral diseases, such as caries and periodontal disease [25]. Mouth breathing has also been correlated, in some studies, to gingivitis and periodontitis, which are associated with chronic skin diseases such as urticaria [25]. An individual who breaths through the mouth presents alterations in the defense mechanisms of oral tissues resulting from exposure to air during respiration, which predisposes gingivitis and the risk of cariogenic activity. Koga *et al*. [42] correlated mouth breathing with the presence of *Streptococcus mutans* in saliva comparing 30 mouth-breathing patients between the ages of six and 11 years old, and 30 nasal breathing children of the same age who had no apparent caries lesions in the oral cavity. The results showed a high prevalence of *Streptococcus mutans* in 70% of mouth breathers and 43.3% of the controls, indicating a higher tendency for cariogenic activity. Yamaguchi *et al*. [29] conducted a study with children (2-6 years old) to verify the association between mouth breathing and atopic dermatitis. These authors used 1036 questionnaires and received 468 replies that indicated that atopic dermatitis was related to mouth breathing in the day and at night [26]. Perugia *et al.* [30], also in 2015, found a high prevalence of mouth breathing in children with atopic dermatitis. These results show that this habit is related to AD characteristics, affects the quality of life of the patients and causes sleep disturbances, especially due to pruritus and urticaria that are caused by the disease [28]. Habits such as mouth breathing and thumb sucking are factors that negatively influence the craniofacial region. Mouth breathing usually alters dental and facial morphology and is associated with allergic conditions such as asthma, rhinitis and atopic dermatitis, among others, while thumb sucking has psychological and behavioral associations [29]. Consequences of these habits, interconnecting their duration, intensity and frequency, are neuromuscular changes that in most cases can produce overexertion (thumb sucking) or open bite, overjet and crossbite (mouth breathing) [29].

Another factor that can cause oral health problems is stress, which has already been considered as one of the triggers for AD exacerbations [11]. These patients may also present psychological stress due to their stigmatization, social isolation and discrimination; factors that may, in turn, contribute to the worsening of the signs and symptoms of the disease [20, 21]. Mizawa *et al*. [28] showed that salivary cortisol levels in AD patients were high when compared to healthy controls, and were related to the severity of AD, according to SCORAD, indicating that they may experience chronic stress.

In addition to psychological stress, mechanical stress can also influence the oral health of patients with AD. This issue is particularly relevant for orthodontic treatment, since the tensile and tension forces promoted in the treatment act as factors of bone remodeling. Some disorders such as allergy and asthma predispose greater root resorption [43, 44]. In an analogous way, it has already been demonstrated, through an animal model, that there is an excessive presence of osteoclasts and root resorption gaps in individuals with cutaneous manifestations of AD after orthodontic movement [45, 46]. Given that several proinflammatory cytokines have already been detected in an orthodontic forced induced resorption process, and that the expression of these factors was found to be higher in the periodontal ligaments of AD mice compared to the controls, where both groups were submitted to tooth movement [46, 47]. Later, these phenotypes were also observed in the dental pulp [46]. In order to elucidate the mechanical basis of the process, an *in vitro* study demonstrated that cells from dental pulp and periodontal ligament that were submitted to stress enhanced secretion of pro-inflammatory factors by interleukin 17- producing T CD4+ lymphocytes [45, 46]. This suggests a key role of dental pulp and periodontal ligament in the process of root resorption induced by orthodontic movement, since the stimulation of osteoclastogenesis mediated by Th17 cells after this mechanical stress [47] has already been demonstrated. Interleukin (IL)-17 is an inflammatory cytokine and an important mediator in root resorption induced by the inflammatory process of orthodontic treatment. This cytokine is present in the mediation of autoimmune diseases such as rheumatoid arthritis, multiple sclerosis and psoriasis and has recently been correlated with the pathogenesis of AD by some authors. Thus, orthodontic movement in AD patients possibly induces greater root resorption, due to the higher levels of Th17 cells in the peripheral blood of these individuals, which demonstrates a direct relation with the severity of the disease [48].

An important factor that may also contribute to the hypothesis that patients with AD have a higher frequency of carious lesions, as observed in the studies of Igawa *et al.* [21] and Perugia *et al.* [30], is that atopic dermatitis promotes dysfunction in the epidermal barrier, consequently decreasing its protective function against allergens in the skin and favoring microbial colonization [30, 49]. Thus, these patients would be more prone to the onset of dental caries which is one of the most common infectious diseases, especially in childhood [49]. Mutations in the Fillaggrin Gene (FLG) may also be associated with the pathogenesis of dental caries, since it has an expression in oral mucosa [50]. Although not all AD patients harbor a mutation in this gene (only 60%), its dysfunction impairs proper formation of the skin barrier. Based in what is observed in filaggrin-deficient skin, it is reasonable that absence of filaggrin in oral mucosa promotes greater susceptibility to dryness and infections caused by microorganisms such as *Streptococcus mutans*, the most common agent of dental caries [30, 51-54].

Based on the articles mentioned above in this review, we have demonstrated a possible relationship between oral health and the condition of the patient with atopic dermatitis, not only from physiological aspects, but also taking into account the microbiological, psychological and individual habits of AD patients.

## CONCLUSION

Some oral manifestations, such as odontogenic infections, candidiasis and mouth breathing, are often diagnosed in patients with atopic dermatitis and may also be directly associated with the worsening of this disease. Thus, careful oral examination in patients with AD should be performed so that the quality of life of these patients can be improved. However, the development of new studies is necessary in order to have a better understanding of oral manifestations and atopic dermatitis.

## Figures and Tables

**Table 1:** Characteristics of some of the studies included in this paper.

**Author, Country and Year**	**Goals**	**Databases**	**Keywords**	**Sample Size**	**Type of Study**	**Factors Associated with Atopic Dermatitis**
Yamaguchi H *et al*. Japan, 2015	To evaluate if mouth breathing is associated with other types of diseases including atopic dermatitis through a questionnaire aimed at pre-school children in day care centers	PubMed; Google Scholar	Atopic dermatitis; mouth	468 children Age: 2-6 years old -59 children with atopic dermatitis; -46 children with asthma; -61 children with allergic rhinitis	Sectional	Daytime and nighttime (during sleep) mouth breathing in children with AD was associated with: history of allergic rhinitis, history of asthma, and family history of atopic dermatitis
Javad G *et al*. Iran, 2015	To verify the colonization of *Candida* and the specific humoral response against *Candida albicans* in patients with atopic dermatitis	PubMed	Atopic dermatitis; mouth; oral pathology	- 100 patients with AD - mean age of 12.1 ± 11.5 years old; - 50 healthy subjects (control group) with a mean age of 39.9 ± 11.45 years old	Sectional	There was no statistically significant difference in relation to: *Candida* colonization in patients with atopic dermatitis and those in the control group; and between the IgM and IgA serum levels of the patients and controls. *Candida* was isolated from the oral cavity of 23% of the patients with AD and 6% of the individuals in the healthy group.
Leibovici V *et al*. Israel, 2007	To determine the presence of *Candida* in the following locations: armpit, tongue and groin of patients with psoriasis and compared to patients with atopic dermatitis and normal controls.	PubMed	Atopic dermatitis; mouth; dental disorders	- 100 adult patients with psoriasis aged from 18 to 84 years old; - 100 adult patients with AD aged from 18 to 83 years old; - 100 normal controls aged from 18 to 80 years old	Sectional	Low prevalence of *Candida* on the tongue of patients with atopic dermatitis (18%) compared to patients with psoriasis (32%) and normal controls (21%).
Igawa K, Nishioka K, Yokozeki H Japan, 2007	To verify if a odontogenic focal infection is an aggravating factor in atopic dermatitis	PubMed	Atopic dermatitis, mouth; dental disorders	- 43 adolescent and adult patients aged from 13 to 62 years old and with chronic AD	Cohort	Patients with AD and an odontogenic focal infection had a greater improvement after 3 months of dental treatment compared to patients who did not have odontogenic focal infection but who received dental treatment.
Mizawa M *et al*. Japan, 2013	To evaluate salivary cortisol levels in patients with atopic dermatitis and to compare them with a healthy control group	PubMed	Atopic dermatitis	- 30 patients with AD aged from 15 to 62 years old; - 42 systematically healthy individuals aged from 31 to 54 years old (group control)	Sectional	The salivary cortisol levels of patients with atopic dermatitis were significantly higher than those of the control group
Perugia C *et al*. Italy, 2015	Verify the possible correlation between atopic dermatitis and oral manifestations in pediatric patients;	PubMed	Atopic dermatitis; caries	300 children (from 2 to 17 years old) - 90 children (30%) with AD; -210 children without AD	Sectional	Children with AD presented: - medical history of spoil habit (76%); - presence of dental caries (56%); - dental malocclusion (64.4%).
